# Novel monoclonal antibodies against thymidine kinase 1 and their potential use for the immunotargeting of lung, breast and colon cancer cells

**DOI:** 10.1186/s12935-020-01198-8

**Published:** 2020-04-16

**Authors:** Edwin J. Velazquez, Taylor D. Brindley, Gajendra Shrestha, Eliza E. Bitter, Jordan D. Cress, Michelle H. Townsend, Bradford K. Berges, Richard A. Robison, K. Scott Weber, Kim L. O’Neill

**Affiliations:** 1 LSB 4007, Department of Microbiology and Molecular Biology, Brigham Young University, Provo, UT 84602 USA; 2 Thunder Biotech, Provo, UT USA

**Keywords:** Thymidine kinase 1, Tumor biomarker, Monoclonal antibody, ELISA, ADCC, Antibody-based therapies

## Abstract

**Background:**

Thymidine kinase 1 (TK1) is a pyrimidine salvage pathway enzyme that is up-regulated in malignant tissues and elevated in the serum of cancer patients. While TK1 has been well established as a tumor biomarker, little has been done to explore its potential as a tumor target. Recently, we reported the membrane expression of TK1 on malignant cells, but not on normal cells. This study explores the possible use of monoclonal antibodies for the targeting of membrane associated TK1 in lung, breast, colon and prostate cancer cells.

**Methods:**

We generated and evaluated a panel of monoclonal antibodies against six different epitopes exposed in the tetrameric form of TK1. Antibodies were developed with hybridoma technology and validated with Western blot, siRNA TK1 knockdown, enzyme-linked immunosorbent assay (ELISA) and flow cytometry. The therapeutic potential of the antibodies was evaluated in vitro in antibody-dependent cell-mediated-cytotoxicity (ADCC) experiments.

**Results:**

Binding of the antibodies to TK1 was confirmed by Western blot in purified recombinant protein, cancer serum, and cell lysate. After a TK1 knockdown was performed, a reduction of TK1 expression was observed with five antibodies. Using indirect ELISA, we identified 3B2E11, 9C10, 7H2, 3B4, 8G2 among the most sensitive antibodies (LOD = 10.73–66.9 pg/ml). Surface expression of TK1 on the membrane of various cancer cell lines was analyzed with flow cytometry. Antibodies 8G2, 3B4, 7HD and 5F7G11 detected TK1 on the membrane of various cancer cell lines, including lung, prostate, colon and breast. No significant binding was detected on normal lymphocytes. Increased cytolysis of lung (~ 70%. *p *= 0.0001), breast (~ 70%, *p *= 0.0461) and colon (~ 50% *p *= 0.0216) cancer cells by effector cells was observed when anti-TK1 antibodies were added during ADCC experiments.

**Conclusions:**

The antibodies developed showed potential to be used to detect and target TK1 on the membrane of various tumor cells. The targeting of TK1 in malignant cells using monoclonal antibodies may be a feasible approach for the elimination of high TK1 expressing tumor cells.

## Background

With the number of clinical and preclinical agents exponentially increasing every year, cancer immunotherapy is currently one of the fastest growing areas in global oncology [1]. From cell adoptive therapies to monoclonal antibodies, the efficacy of most cancer immunotherapies primarily relies on the discovery of suitable tumor targets and the development of highly specific agents against these targets [2]. During the last decade the list of tumor antigens available for immunotherapy have gone from dozens to hundreds, allowing us to treat a broader spectrum of human malignancies [3]. However, a common limitation that many of these tumor targets face is their expression on normal tissues [4]. Thymidine Kinase 1 (TK1) is a cell cycle regulated DNA synthesis enzyme that is up-regulated in malignant tissues during early stages of cancer development [5, 6]. Multiple studies have shown that TK1 levels in serum (sTK1) and tissues correlate with cancer progression, patient outcome and recurrence events [7–12]. Although TK1 was initially proposed as a cancer biomarker for several blood cancers [13, 14], it has also been shown to be a reliable biomarker for a wide variety of solid malignancies [15–20].

While TK1 levels have been primarily used to monitor the development of malignancy, it has been suggested that overexpression of TK1 or malignant associated forms of the enzyme could be used for the targeting of cancer [21, 22]. Recently, the expression of membrane associated TK1 forms in both cancer cell lines and clinical samples has been reported. In one study it was shown that monomeric and dimeric forms of TK1 can be detected on the cell membrane of mononuclear cells (MNC) from patients with acute lymphoblastic leukemia (ALL) and acute myeloid leukemia (AML) [23]. While this membrane associated TK1 form was present on malignant cells, it was absent on normal proliferating B cells [23]. According to another study, membrane expression of TK1 was also found in lung cancer cell lines and cells from breast and colon tumors [24]. These findings indicate that TK1 may be a potential immunotherapeutic target for antibody-based and adoptive cell therapies.

Despite the large number of studies demonstrating the value of TK1 as a cancer biomarker and its potential as a tumor target, there are a limited number of clinically tested antibodies for the detection and targeting of TK1 [25]. To our knowledge, no TK1 antibody-based therapeutics have been developed or tested in a preclinical setting yet [26]. Moreover, most of the existing antibodies for detection of TK1 primarily target a cell cycle regulatory region of the TK1 molecule at the C-terminus [27]. It has been reported that TK1 in malignant cells has different isoenzymes and that its activity levels differ significantly from TK1 in normal cells [28, 29]. It is also known that TK1 can form different complexes. Some of these complexes in serum having abnormal molecular weights of up to 240 kDa and 730 kDa [30–33]. Therefore, antibodies targeting only one region of the TK1 protein may limit the detection of TK1 to specific forms of the enzyme. Thus, the existing antibodies for TK1, may not be sufficient to detect some of the complex TK1 forms that exist in cancer patients. Broadening the spectrum of targetable TK1 specific epitopes could help us increase our ability to target TK1 in cancer patients.

In this study, we identified six epitopes that were exposed in the tetrameric form of TK1 and generated monoclonal antibodies specific for these regions. Seventeen antibodies were chosen for characterization and validation through Western blot, siRNA TK1 knockdown, ELISA and flow cytometry. In addition, the potential of the anti TK1 antibodies for the targeting of malignant cells was tested by measuring the antibody-dependent cell-mediated cytotoxicity (ADCC) responses of mono nuclear cells (MNC) against high-level TK1-expressing cancer cells.

## Methods

### Cell lines and isolation of MNC

The NCI-H460 (ATCCHTB-177™), A549 (ATCC^®^ CCL-185), MDA-MB-231 (ATCC^®^ CRM-HTB-26), PC3 (ATCC^®^ CRL-7934™) and HT-29 (ATCC^®^ HTB-38™) cell lines were obtained from the American Type Culture Collection (ATCC, Manassas, VA, USA) and maintained according to ATCC recommendations. A549, NCI-H460, PC3 and HT-29 were cultured in RPMI-1640 media (ThermoFisher scientific, Waltham, MA) supplemented with 10% fetal bovine serum (FBS) and 2 mM l-Glutamine. MDA-MB-231 cells were cultured in DMEM with 2 mM l-glutamine and 10% FBS. Cell lines were grown in an incubator at 37 °C and 5% CO_2_. All cell lines used were tested for TK1 surface expression with flow cytometry with a commercial antibody Abcam (91651) (Abcam, Cambridge, UK) to confirm the presence of TK1 on the cell membrane. Human MNC were isolated from whole blood from healthy donors with the high affinity CD16 V158 FcγIIIa receptor variant [34]. The CD16 V158 FcγIIIa variant was confirmed by polymerase chain reaction (PCR) from cDNA of the donor’s MNC and DNA sequencing. MNC were isolated with lymphocyte separation media (Corning, NY) following manufacturer instructions, red blood cells were depleted with red blood cell lysis buffer (Biolegend, San Diego, CA) and the MNC were resuspended in RPMI media with 2 mM l-glutamine and 10% heat inactivated human AB serum (Millipore SIGMA, St Louis, MO, USA). Blood withdrawal was done under the Brigham Young University’s institutional review board approval No. 1734.

### Production of recombinant TK1 in *E. coli* and *Saccharomyces cerevisiae*

The production of human TK1 was carried out in an *E. coli* expression system by Genscript’s recombinant protein service. In-house production of human TK1 was done using the pESC-URA (Genscript, Piscataway, NJ) yeast expression system and a *Saccharomyces cerevisiae* yeast strain with a REG-1 mutation. Briefly, the coding sequence of the human TK1 (Genbank NM_003258.5) was synthesized and placed into the pESC-URA vector flanked by the Sal I restriction sites. A tag of 6 histidines was included at the C-terminus of the TK1 sequence to facilitate His-tag purification. The TK1-pESC-URA vector was introduced in electrocompetent yeast using the lithium acetate procedure and an Eporator system (Eppendorf, Hamburg, Germany) [35]. After electroporating, the cells were plated in synthetic complete (SC) drop-out Ura-plates (Takara Bio USA Inc, Mountain View, CA) and grown for 36 h at 30 °C. Yeast culture was scaled up to 500 ml and induced for protein expression with galactose. After 36 h, yeast was harvested and brake-open in lysis buffer with the Halt™ protease inhibitor cocktail (ThermoFisher Scientific, Waltham, MA) in a French press. Recombinant TK1 was purified from cleared lysate using NI-NTA-agarose beads columns (Qiagen, Hilden, Germany) and validated with commercially outsourced TK1 produced in *E. coli* by Genscript and the commercial anti TK1 antibody ab91651 in Western blot.

### Epitope selection

Epitopes that could be accessible to antibodies in the active form of the TK1 enzyme, were determined analyzing the 1XBT crystal structure of the tetrameric form of TK1 using the PyMOL software [36, 37]. The epitope sequences were then analyzed using the protein BLAST tool from NCBI with the non-redundant protein sequences and the *Homo sapiens* (taxid9606) data bases to see the epitopes’ similarity with other human proteins. The sequences of the mouse, rabbit, dog and human TK1 isoform 1 (Genebank, NM_003258.5) were aligned and analyzed using the Geneious software to identify regions across the human TK1 sequence that significantly differ between species [38].

### Production of hybridomas and selection of antibody clones

Antibodies were generated in mice and rats that were immunized with 6 different TK1 peptide sequences that were selected as described in the previous section. The peptide sequences for TK1 and the hybridoma cell lines were produced using the monoclonal antibody generation service MonoExpress™ Premium (Genscript, Piscataway, NJ). Briefly, the production of hybridomas consisted in four phases as follows. Phase one consisted in the preparation of the immunogen. In this case the synthesis of six TK1 peptides using the PepPower™ peptide synthesis service (Genscript, Piscataway, NJ). Phase two consisted in the immunization of 3–5 Balb/c mice or rats with the MonoExpress™ immunization protocol. After the immunization regimen was completed, splenocytes were isolated and fused to myeloma cells using polyethylene glycol (PEG) and electrofusion. The cells were then cultured in hypoxanthine-aminopterin-thymidine medium (HAT) to select only the myeloma-lymphocyte hybrids. During phase three, individual hybridoma cells were isolated through limiting dilutions and their supernatants were tested for binding to 1 μg/ml of each TK1 peptide used for immunization by indirect enzyme-linked immunosorbent assay (ELISA). The ten hybridomas with the best screening results were then selected for isotyping. The supernatants were then sent for in-house testing for binding to TK1 in Western blot. Phase four, after antibody binding to TK1 was confirmed through indirect ELISA and Western blot each respective hybridoma was subcloned by the limiting dilution technique, expanded and frozen. From 44 hybridomas a total of 17 clones were selected based on Western blot and Indirect ELISA results. The monoclonal antibodies were then purified from hybridoma supernatant with protein G and protein L columns (ThermoFisher scientific, Waltham, MA) and eluted in phosphate-buffered saline (PBS) buffer.

### Western blot

Western blot was performed to validate the binding of the custom antibodies to TK1 using recombinant human TK1 produced with a yeast expression system to mimic the TK1 folding in human cells. The antibodies were also tested for their capacity to bind to TK1 in serum samples from lung cancer patients. Briefly, 0.5 μg of TK1 or 15 μg of serum samples were mixed with 6× Laemmli buffer (Millipore SIGMA, St Louis, MO). The protein samples were then heated at 100 °C for 5 min and loaded into a 12% SDS-PAGE. The proteins from the gel were then transferred to nitrocellulose membranes (Bio-Rad, Hercules, CA, USA). After blocking with 5% milk in PBS tween-20 (PBS-T) buffer for 1 h at room temperature the blocking solution was poured out and primary antibody solution (1–2 μg/ml) was added. Membranes were incubated at 4 °C overnight. After overnight incubation membranes were washed 3 times with PBS-T buffer. The bound proteins were detected using a 1:20,000 solution of horseradish peroxidase-conjugated (HRP) anti mouse or anti rat antibody (Advansta Corporation, San Jose, CA). The proteins were then detected through the peroxidase reaction using enhanced chemiluminescence (ECL) (Advansta Corporation, San Jose, CA). Films were exposed for different amounts of time depending on the antibody being tested, times ranged from 30 s to 5 min. The films were scanned, and the images were analyzed using the software ImageJ from NIH [39].

### siRNA TK1 knockdown

For our TK1 siRNA experiments we used the validated TK1 siRNA s14160 Silencer^®^ Select (ThermoFisher Scientific, Waltham, MA). The Silencer™ GAPDH (Cat. No. 4390849) siRNA was used as positive control and the Silencer™ siRNA control No. 1 (Cat. No.4390843) was used as negative control. A total of 2.5 × 10^5^ MDA-MB-231 cells were seeded in a 6 well plate and incubated overnight at 37 º C and 5% CO_2_. Cells were then transfected using 7.5 μl of lipofectamine RNAiMAX (Invitrogen, Carlsbad, CA) and 30 pmol of each corresponding siRNA following the manufacturer's protocol. After 24 h transfection was repeated. Cells were harvested after 24 h after second transfection for subsequent experiments and cell lysate preparations. Cell lysates were prepared using NP-40 cell lysis buffer with Halt™ protease inhibitor cocktail (100×) (ThermoFisher Scientific, Waltham, MA) and 1 mM of phenylmethylsulfonyl fluoride (Millipore SIGMA, Burlington, MA). Lysates were cleared by centrifugation and flash frozen with liquid nitrogen and stored at − 80 °C. The cell lysates were then analyzed with Western blot comparing cell lysates treated with TK1 siRNA with the GAPDH siRNA and siRNA negative controls for six custom TK1 antibodies and the commercial TK1 antibody Ab76495 (Abcam, Cambridge, UK).

### ELISA

The sensitivity of each antibody to detect TK1 protein was estimated by indirect ELISA. In order to determine the limit of detection (LOD), antibodies were tested with individual dose–response curves and a four-point parameter logistic curve (4PL). Costar 96-well plates (Corning, NY, USA) were coated overnight with 100 μl of 1:1 serial dilutions of TK1 in PBS buffer at 4 °C. Dilutions were ranging from 5000 to 26 ng/ml or from 6000 to 5 pg/ml depending on the antibodies’ sensitivity. After overnight incubation, the plates were washed 3 times with PBS-T and blocked for 1 h with PBS 1% BSA at room temperature on a shaker with gentle agitation. The plates were then washed 3 times with PBS-T and 100 μl of a 1 μg/ml solution of primary antibody were added into each well. The plates were incubated for 1 h at room temp on a shaker with gentle agitation. After incubating the plates were washed 3 times with PBS-T and 100 μl of a 1:40,000 dilution of secondary HRP-conjugated antibody (Advansta Corporation, San Jose, CA) solution were added into each well. Plates were incubated for 1 h at room temperature on a shaker. The plates were washed 3 times with PBS-T, and 100 μl of 1-step ultra TMB substrate (ThermoFisher scientific, Waltham, MA) were added to each well. The plates were then incubated for 10 min at room temperature, protected from light. The reaction was stopped by adding 100 μl of 0.5 M sulfuric acid solution. The absorbance was measured using a Synergy HT Microplate Reader (Bio-Tek Winooski, VT) at 450 nm and 650 nm. Samples were run in triplicate and confirmed with at least two independent experiments.

From the antibodies that showed high sensitivity in indirect ELISA mμltiple antibody pairs were tested in sandwich ELISA format. One hundred μl of a 0.5 μg/ml solution of the capture antibodies were allowed to adsorb overnight at 4 °C. After overnight incubation, the plates were washed 3 times with PBS-T and blocked with PBS 1% BSA as previously described. TK1 protein or cancer serum was serially diluted in PBS buffer and then allowed to bind to the primary antibody for 1 h at room temperature on a shaker. The wells were then washed 3 times with PBS-T buffer and 100 μl of biotinylated detection antibody solution (0.5 μg/ml) was added and allowed to bind for 1 h at room temperature. After incubation, the plates were washed with PBS-T buffer and 100 μl of Pierce high sensitivity streptavidin-HRP solution (ThermoFisher scientific, Waltham, MA) was added to each well. The plates were incubated for 30 min. and washed with PBS-T buffer 3 times. After washing, 100 μl of 1-step ultra-TMB substrate solution (ThermoFisher scientific, Waltham, MA) was added to the wells and the plates were incubated with the substrate for 10 min. The reaction was quenched by adding 100 μl of 1 M sulfuric acid. Absorbance was measured using a Synergy HT Microplate Reader (Bio-Tek Winooski, VT) at 450 nm and 570 nm.

For each antibody the limit of quantification (LOQ) and the LOD were determined in ELISA and using the following formulas, LOQ = 10 (*s/f*ʹ*)* and LOD = 3.3(*s/f*ʹ) [40, 41]. Where *s* is the standard deviation from the residuals and *f’* is the slope of a calibration curve. A 30% maximum value for relative standard deviation for the concentration estimates was set for the LOD and a 10% for the LOQ, according to Hayashi et al.

### Flow cytometry

Surface expression of TK1 was analyzed through flow cytometry. The antibodies binding capacity to detect membrane associated forms of TK1 was compared to the binding capacity of the commercial TK1 antibody Ab91651 and Ab76495 (Abcam, Cambridge, UK). For the experiment four different cancer cell lines expressing different levels of TK1 were used. The cell lines used were the non-small cell lung carcinoma cell line NCI-H460, the breast cancer cell line MDA-MB-231, the prostate cancer cell line PC3 and the colorectal cancer cell line HT-29. Since membrane expression of TK1 appears to be restricted to malignancy, MNC from healthy donors were used as a negative control. Each one of the cancer cell lines was stained with each one of the anti TK1 antibodies in at least 3 independent flow cytometry experiments.

For the analysis cancer cells and normal MNC were collected as follows. Cancer cells were grown as monolayer cultures using DMEM or RPMI1640 with 10% FBS and 10 mM l-glutamine. The cells were grown until they reached 70–80% confluency and detached using accutase (ThermoFisher scientific, Waltham, MA). The cells were then collected and washed 3 times with cell staining buffer (PBS buffer, pH7.4, BSA 1% and sodium azide 0.1%). MNCs were isolated from whole blood as previously described and washed 3 times with cell staining buffer. All cells were resuspended at a concentration of 5 × 10^5^ cells/ml, placed in individual microcentrifμge tubes and incubated with 5 μl of Human TruStain Fc block (BioLegend, San Diego, CA) for 10 min at room temperature. In the case of MNC, the cells were incubated with 10% heat inactivated human serum 30 min on ice. Unstained, isotype, and positive controls (CD44, CD45, GAPDH) were included in all experiments. After incubation with Fc block the cells were stained with 1 or 2 μg of the custom and commercial TK1 antibodies and incubated for 30 min on ice. The cells were then washed 3 times and resuspended in 100 μl of cell staining buffer. Secondary antibodies conjugated to Alexa Fluor 647 (Abcam, Cambridge, UK) were added and the cells were incubated on ice for another 30 min, protected from light. After incubating with the secondary antibodies, the cells were washed 3 times, resuspended in 100 μl of cell staining buffer and analyzed. Dead cell discrimination was performed adding 15 μl of a 10 μg/ml PI solution (Millipore SIGMA, St Louis, MO) 1 min before analysis. A Cytoflex flow cytometer machine (Beckman Coulter, Brea, CA) was used to collect the data. The data analysis was performed using FlowJo software (FlowJo, Inc., Ashland, OR).

### Detection of membrane associated TK1 and isolation of plasma membrane protein

To further confirm the levels of membrane associated TK1 from cancer and normal cells, we isolated plasma membrane protein fractions from cancer and normal cells. For this experiment the non-small cell lung cancer cell line NCI-H460 was chosen for presenting high levels of membrane associated TK1. MNCs from a healthy individual were used as normal cells. To isolate the plasma membrane protein we used the extraction kit (Abcam, Cambridge, UK) and followed the manufacturer instructions. Briefly 2 × 10^8^ cells were harvested and gently lysated with a Dounce homogenizer in 2 ml of lysis buffer with protease inhibitors. After lysis, the cells were centrifuged at 700*g* for 10 min at 4 °C and the supernatants were transferred to a new tube. The cleared supernatants were then centrifuged at 10,000*g* for 30 min at 4 °C. The supernatant containing the cytosolic fraction was completely aspirated and the cell pellet containing the membrane protein fraction was resuspended and mixed with upper and lower phase solutions as instructed from the manufacturer. The total plasma membrane protein was then quantified using the BCA assay kit (ThermoFisher scientific, Waltham, MA). A Total of 30 μg of protein was loaded into a SDS-PAGE gel per each sample. The SDS-PAGE, transfer of the protein to a nitrocellulose membrane and Western blot were performed as previously described. To detect TK1 we used a validated TK1 antibody. As negative control we used an anti-GAPDH antibody (Cell Signaling, Danvers, MA) and as positive control we used an anti-sodium/potassium ATPase antibody MA3-928 (ThermoFisher scientific, Waltham, MA).

### Antibody-dependent cell-mediated cytotoxicity experiments

The capacity of the custom antibodies to elicit ADCC responses was tested in vitro. For these experiments the A549 cell line which expresses high levels of TK1 on the cell membrane was chosen as a target cell. To measure cell death overtime, we used the real time cell analysis platform ExCELLigence (ACEA biosciences, San Diego, CA). The ExCELLigence platform works based on the principle of impedance. Tissue culture plates with nanogold electrodes were used to measure impedance caused by target cells adhering to the plates. The impedance reflects cell growth and cell viability. The cell growth is then expressed as a normalized cell index (NCI) that is measured overtime. The higher the cell index (CI), the more cell growth. A decrease in the cell index reflects cell death or cytotoxicity caused by a treatment, in this case, an ADCC response [42]. For the ADCC experiments a max of 8 × 10^3^ target cells were seeded in each well in a total volume of 100 μl of RPMI media. Experiments were run for 48–72 h CI were normalized to 1 at the time when effector cells and antibodies were added. After sufficient growth (CI 0.5–1.0) 50 μl of freshly isolated MNC cells were added at different effector-target ratios (E:T). E:T ratios of 5:1, 2.5:1, 1.25:1 and 0.625:1 were tested. After finding the optimum E:T ratio, dose response curves were generated by testing different concentrations of the TK1 antibodies to find the minimum amount of antibody that could produce a significant increase in specific cell death. All experiments included cells treated with the IgG2b negative control antibody (BIO-RAD, Hercules, CA). Experiments also included controls of target cells without antibody and a full cell lysis control. The effect of the TK1 antibodies in target and effector cells alone were also tested and included as additional controls. The percentage of specific cytolysis was determined using the immunotherapy module from the RTCA pro software (ACEA biosciences, San Diego, CA). The signal from effector and target cells with no antibody and from effector alone was subtracted. According to the user’s manual the software subtracts the values from the reference wells using the following formula:

$$\% {\text{ Cytolysis}}_{\text{st}} = \, [ 1- {\text{Norm}}({\text{CI}}_{\text{st}} - {\text{AvgEff}}_{{\_{\text{Sub}}\_{\text{t}}}} )/({\text{AvgNorm}}({\text{CI}}_{\text{Rt}} - {\text{AvgEff}}_{{{\text{R}}\_{\text{Sub}}\_{\text{t}}}} )] \times 100$$

where Norm (CI_st_-AvgEff_*_Sub_t*_) is the Normalized value of the sample Cell Index minus the Av-erage Cell Index for the matching selected Effector alone control at time *t* and AvgNorm(CI_*Rt*_- AvgEff_*R_Sub_t*_) is the average normalized cell index value of the reference wells minus the Average Cell Index of the matching effectors used in the reference at time *t*. If target alone is used as reference, there is no subtraction of AvgEff*R_Sub_t* [43].

To add a qualitative element to our ADCC experiments and visually monitor the ADCC responses of MNCs against different target cells we also used the ImageXpress^®^ Pico system (Molecular devices, San Jose, CA). For this assay we choose two cell lines positive for TK1 on the cell membrane, the MDA-MB-231 and the HT-29 cell lines. To allow the ImageXpress^®^ Pico system to count individual cells, the cells were engineered with the IncuCyte^®^ Nucligth Green Lentivirus reagent (Sartorius, Gotinga, Germany) to express nuclear restricted green fluorescent protein (Nuc-GFP). Cells were seeded at the same density described above and grown overnight under controlled environmental conditions (37 °C, 5% CO_2_). Effector cells and anti TK1 Antibodies were then added to the target cells and the target cells were counted each hr for 48–72 h. Normalized GFP cell count was compared between treated and untreated cells. Antibody isotype controls were included as negative controls.

### Statistical analysis

All the statistical analyses were performed using GraphPad Prism software (GraphPad, San Diego, CA). For the analysis of the dose–response curves from the indirect ELISAs, the data were log-transformed and analyzed with a 4-parameter non-linear regression analysis with a 95% confidence interval (CI). The slope, the R square, and standard deviation of the residuals were calculated. A test for goodness of fit, replicates test for lack of fit and test for homoscedasticity were performed. For ADCC experiments involving dose–response curves, two-way repeated measures ANOVA analyses were performed to compare different treatments during a course-time. Analysis of multiple comparisons was performed comparing the mean of each treatment with every other treatment mean. Correction of multiple comparisons was done with the Tukey test. For cytolysis experiments isotype control vs treatment group were compared using multiple two tailed T-tests. Non-consistent standard deviation was assumed.

## Results

### Production and validation of recombinant human TK1

About 2 mg/ml of soluble protein was obtained from a 500 ml liquid culture. In-house produced TK1 in yeast was then validated with Western blot using commercially outsourced TK1 (< 80% pure) produced by Genscript in an *E. coli* expression system as positive control. A band of 25 kDa can be observed in both TK1 sources, commercially outsourced (bacteria) and in-house produced (yeast) (Additional file 1) TK1 was detected with the anti-TK1antibody ab91651. Thus, validating our TK1 production process. In addition, a 50 kDa dimer band was observed in the TK1 produced in our yeast expression system showing the ability of our in-house TK1 to properly fold and make TK1 complexes.

### Selection of targetable regions in the active form of TK1

**Fig. 1:**
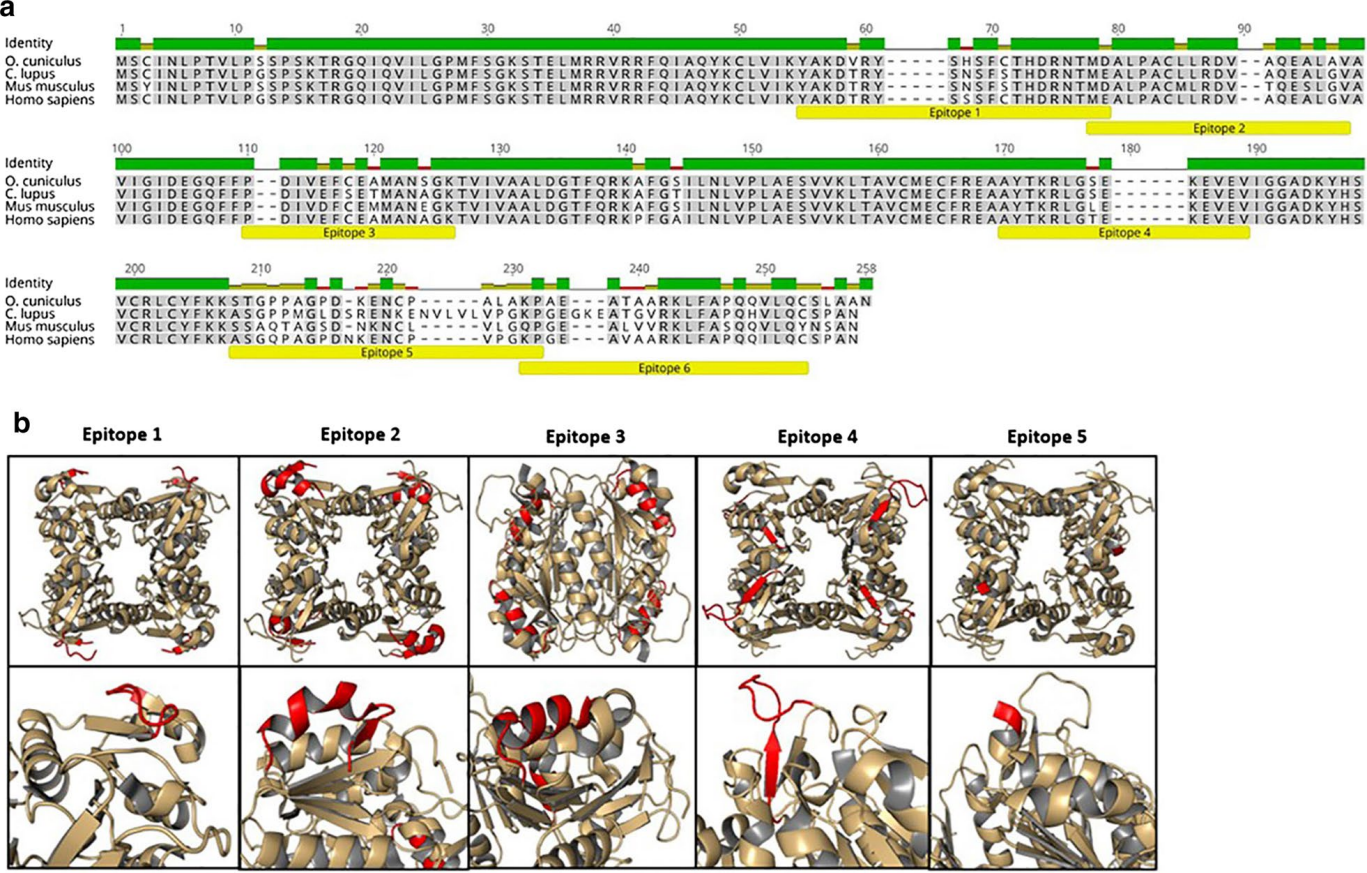
**a** Alignment of the amino acid sequences of thymidine kinase 1 from 4 different species, *Canis lupus familiaris*, *Oryctolagus cuniculus*, *Mus musculus and Homo sapiens*. The conserved regions are highlighted and the differences between species are shown in the identity top bar. TK1 epitopes are annotated in yellow. **b** The tetrameric form of TK1 was 3D modeled using the PyMOL software and the 1XBT crystal structure from the NCBI protein data bank. The Peptide sequences utilized in the generation of monoclonal antibodies were highlighted in red. The epitopes selected for antibody generation were all peptide sequences that were exposed and hypothetically accessible to antibody binding. Epitope 6 is localized at the end of the C-terminus of the TK1 molecule but not visible in the available crystal structures of TK1

To identify regions specific for human TK1, alignments of the human TK1 sequence with those of mice, dog and rabbit sequences showed conserved and variable regions among species. However, the epitopes of our interest were localized in the most variable regions of the TK1 human sequence when compared with the other species (Fig. 1a). The similarity of the human TK1 sequence to other species is shown in Table 1. Six different epitopes across the TK1 molecule were chosen for antibody generation (Table 2). The epitopes covered accessible regions in the tetrameric form of the human TK1 isoform 1, from the N-terminus to the C-terminus (Fig. 1b). BLAST results showed 100% coverage and identity for most of the epitopes’ hits specific for TK1 protein with E values ranging from 10^−5^ to 10^−15^. We set an E value of 1e^−6^ as a tolerance value for all the hits the epitopes had as recommended by the NCBI BLAST QuickStart tutorial [44]. We did not see any non-TK1 related hits equal or below the cut-off value from the six epitope sequences. Thus, the epitope sequences seemed to be unique to the human TK1 and no significant similarities with other human proteins were found.

**Table 1:**
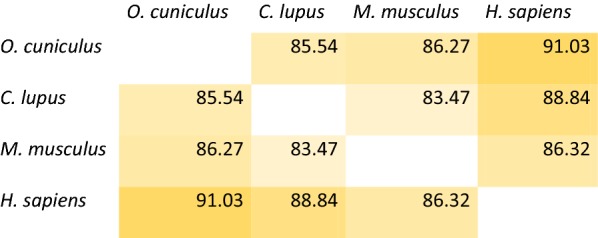
Percentage of similarity between the TK1 sequences of different animal species

**Table 2:** The amino acid sequences and regions of the selected TK1 epitopes

Epitope	Sequence	Region
1	CYAKDTRYSSSFCTHDRNTME	55–74
2	MEALPACLLRDVAQEALGVC	73–91
3	CPDIVEFCEAMANAGK	103–117
4	CAYTKRLGTEKEVEV	161–174
5	CASGQPAGPDNKENCPVPGKP	193–212
6	KPGEAVAARKLFAPQQILQC	211–230

### Validation of TK1 antibodies through western blotting

**Fig. 2:**
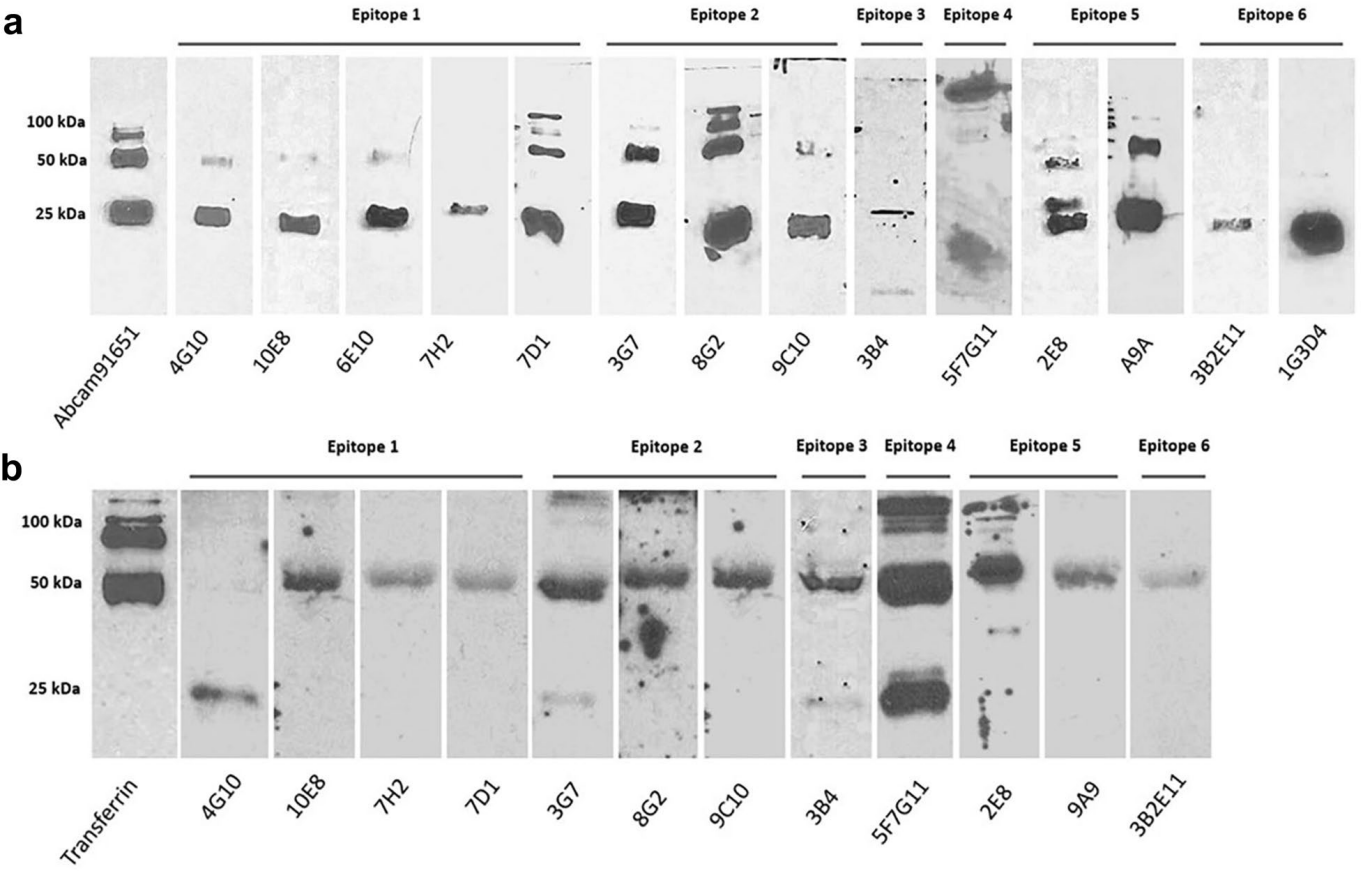
Western blot analysis of the generated antibodies for all 6 epitopes. The antibodies capacity to detect human TK1 was tested. **a** An amount of 0.5 μg of recombinant human TK1 was loaded in an SDS-PAGE and transferred to nitrocellulose membranes. **b** An amount of 20 μg of serum from a stage IV lung cancer patient was loaded in each lane. The membranes were probed with the custom TK1 antibodies and compared with a commercial TK1 antibody. For serum a transferring antibody was included as a serum loading control and to test serum integrity

An initial screening using supernatants from the hybridomas clones was conducted to identify antibody candidates. After this initial screening a panel of 17 hybridoma clones were selected for antibody production. To determine if these antibodies had the capacity to bind human TK1 protein, we first performed western blotting with purified recombinant TK1 from our yeast expression system and compared each of the antibodies with the commercial TK1 antibody Abcam (91651). The results showed that all antibodies except 10H2, 6A11, and 8F12 showed binding to the recombinant purified TK1 protein (Fig. 2a). It is known that TK1 exists in multiple forms such as the monomeric, dimeric, and tetrameric form. We found that antibodies 4G10, 10E8, 6E10, 7D1, 8G2, 2E8 and 9A9 were able to bind multiple forms of TK1 including the dimeric and tetrameric forms. In addition, the majority of the antibodies demonstrated ability to detect TK1 in the serum of cancer patients (a representative sample using serum from a stage IV lung cancer patient is shown in Fig. 2b). Although the antibodies mainly detected the dimeric form of TK1 in serum, we observed that antibodies 4G10, 3G7, and 3B4 were able to detect the TK1 monomeric form while the antibodies 5F7G11 and 2E8 seemed to detect a broader range of TK1 complexes from 25 kDa to 100 kDa or larger forms.

### Antibody validation with a siRNA TK1 knockdown

**Fig. 3:**
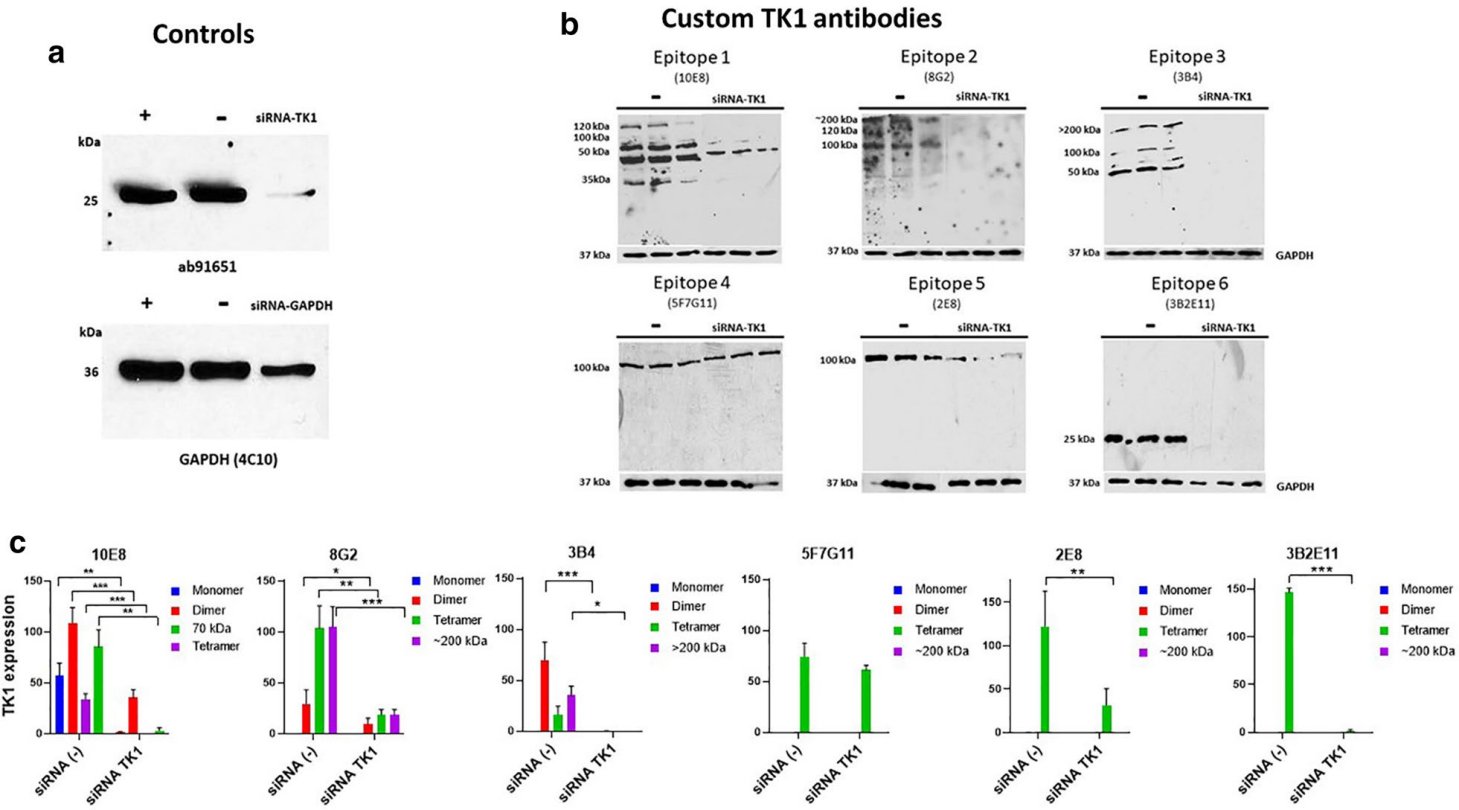
Validation of the custom anti-TK1 antibodies with a TK1 siRNA knockdown experiment in MDA-MB-231 cells. **a** GAPDH positive, SiRNA negative controls and TK1 siRNA s14160 were tested with commercial anti GAPDH and anti TK1 antibodies. **b** Validation of 6 custom TK1 antibodies with Western blot analyzing cell lysates from siRNA negative control and TK1 siRNA treated MDA-MB-231 cells. **c** Normalized TK1 expression in reference to GAPDH and comparison of expression levels between cells treated with TK1 siRNA and untreated

After having confirmed the binding of the custom antibodies to recombinant TK1 and TK1 in human serum samples. We decided to validate the specificity of six antibodies by silencing the expression of TK1 using TK1 siRNA. In order to do that we first tested if we were able to successfully knockdown TK1 in the breast cancer cell line MDA-MB-231. We observed a significant reduction in the expression of TK1 in the cells treated with the TK1 siRNA while expression of TK1 using GAPDH and the non-specific siRNAs was significantly higher. Similarly, the cells treated with GAPDH siRNA showed a decrease of GAPDH expression according to Western blot analysis. GAPDH expression was not affected by TK1 siRNA or negative siRNA control (Fig. 3a).

Six different antibodies against the six different TK1 epitopes were tested including antibodies: 10E8 (Epitope 1), 10H2 (Epitope 2), 3B4 (Epitope 3), 5F7G11 (Epitope 4), 2E8 (Epitope5) and 3B2E11 (Epitope 6). Each antibody was tested using cells lysate from cells treated with a negative siRNA control compared with a cell lysate from cells that were treated with TK1 siRNA. A significant reduction in the detection of TK1 was noticed with antibodies 10E8, 8G2, 3B4, 2E8 and 3B2E11. This difference was particularly pronounced with antibody 3B2E11 which is the most sensitive antibody clone. No difference in the TK1 expression between cell lysates was observed with antibody 5F7G11 (Fig. 3b, c). We observed that the main forms of TK1 present in cell lysate were the dimeric and the tetrameric forms. In the case of antibodies 10E8, 8G2 and 3B4 we could also observe TK1 forms of high molecular weight around or above 200 kDa, such bands were not at detectable levels in cell lysate of siRNA TK1 treated cells. While antibodies 10E8, 8G2 and 3B4 were able to detect multiple forms of TK1 in cell lysate, antibodies 2E8 and 3B2E11 detected only tetrameric and monomeric forms respectively.

### Antibody validation through ELISA, targeting of different TK1 regions and sensitivity of the antibodies

**Fig. 4:**
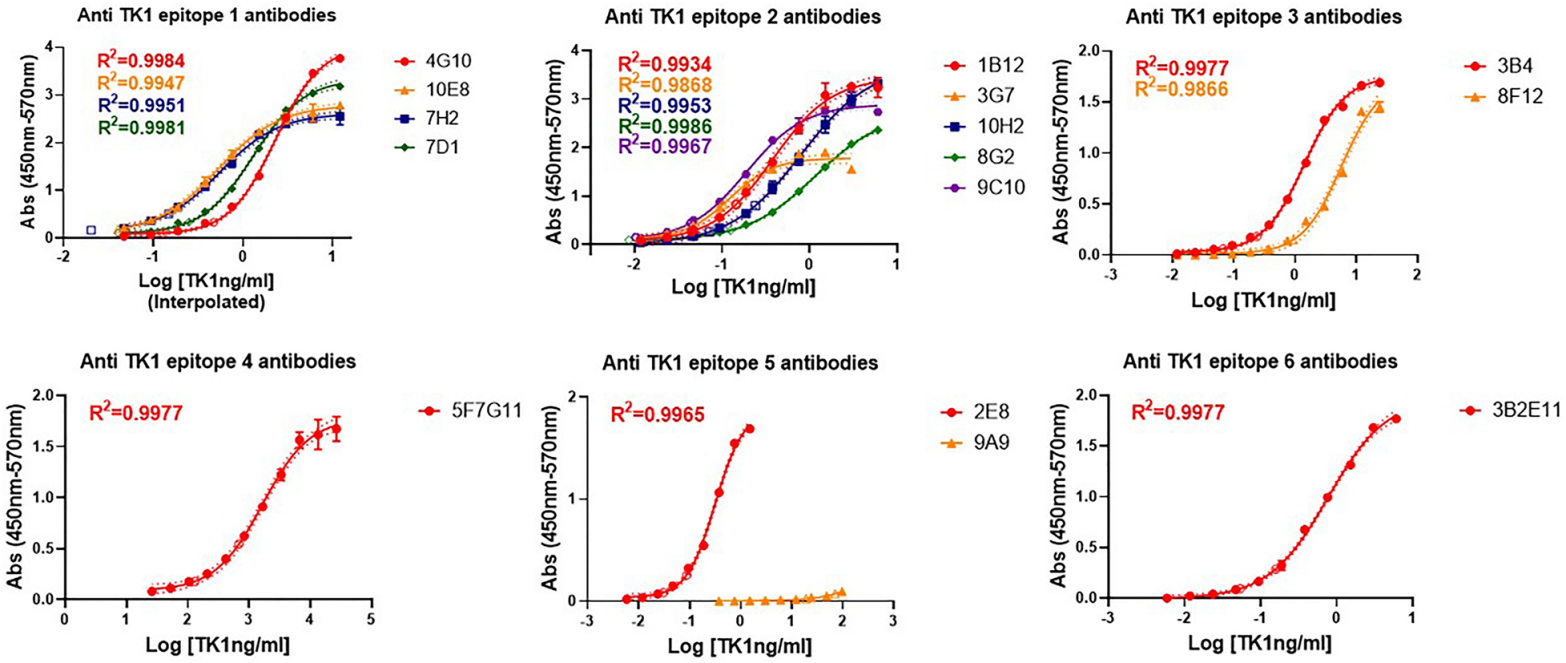
Calibration curves of indirect ELISAs. Each antibody’s binding capacity to TK1 was tested in indirect ELISA. A 4-point non-linear regression analysis was performed for each dose–response curve. Antibodies graphs are combined per epitope

To find the most sensitive antibodies for the detection of TK1, each of the antibodies was first individually tested by indirect ELISA using a dose–response curve (Fig. 4). The curves were then analyzed with a 4-parameter non-linear regression and the limit of detection (LOD) of each antibody was determined. From the 17 antibodies 13 fitted the shape of a sigmoidal curve and passed the goodness of fit test. The R squared values ranged from 0.9696 to 0.9989. For the clone 9A9 we could see the bottom and the steepest part of the curve but not the top plateau phase. This was due to the available range of the TK1 dilutions. However, a slope could be calculated with the linear portion of the curve. Antibody clones 6A11 and ID3G4 did not develop a detectable signal in ELISA. Thus, were excluded from further testing. For 15 of the 17 TK1 antibodies the inter-assay CV% was between 1.52 and 17.32, while the intra-assay CV% was between 0.45 and 11.40.

**Fig. 5:**
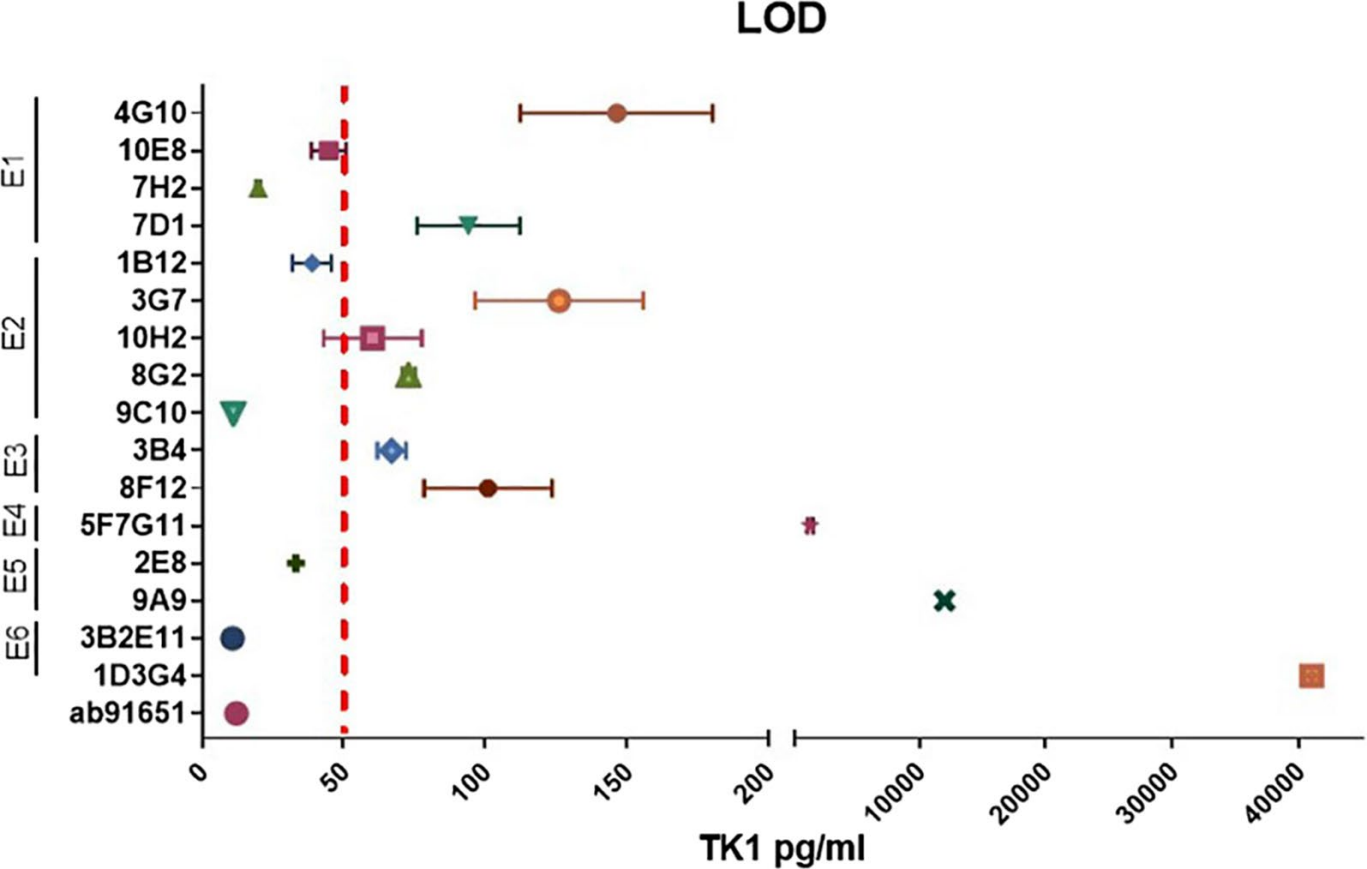
A comparison of antibody sensitivities. The LOD values of each antibody were plotted and compared. The most sensitive clones were 3B2E11, 9C10, 7H2, 2E8, and 1B12

The limit of detection was calculated for each of the antibodies as previously described in the methods section. The interpolated values, standard deviations and percentage of the inter and intra coefficient of variance for each antibody are showed in Table 3. From the indirect ELISA, we found that the most sensitive antibodies were produced by the clones 3B2E11, 9C10, 7H2, 2E8, and 1B12 with clone 3B2E11 producing the most sensitive antibody, having a LOD between 8.87 and 12.58 pg/ml (Table 3 and Fig. 5). These antibodies were able to detect concentrations of TK1 in the picomolar range thus making them candidates for clinical and therapeutic applications.

**Table 3:** Sensitivity of the TK1 antibodies

Clone	ID	Isotype	Host	Mean	SD±	Inter CV	Intra CV
				LOD pg/ml	LOD	%	%
4G10	1-1	IgG2a, κ	Ms	146.40	33.96	16.4	5.72
*10E8	1-2	IgG1, κ	Ms	44.67	4.33	9.69	11.40
*7H2	1-7	IgG1, κ	Ms	19.79	0.55	2.78	9.71
7D1	1-9	IgG1, κ	Ms	94.03	12.86	13.68	8.41
*1B12	2-1	IgG2a, κ	Ms	38.76	4.84	12.5	4.57
3G7	2-6	IgG2a, κ	Ms	125.96	21.0	16.67	1.09
10H2	2-15	IgG2a, κ	Ms	56.50	15.96	20.3	8.2
*8G2	2-17	IgG2b, λ	Ms	72.87	4.83	1.62	8.38
*9C10	2-19	IgG2a, κ	Ms	11.01	0.67	6.1	4.0
*3B4	3-5	IgG2b, κ	Ms	66.90	3.5	5.33	0.45
6A11	3-6	IgG1, κ	Ms	NA	NA	NA	NA
8F12	3-10	IgG1, κ	Ms	101.00	16	15.84	2.5
5F7G11	4-2	IgG2a, λ	Rat	1376.58	153.42	11.14	0.71
*2E8	5-1	IgG2b, λ	Ms	33.10	0.50	1.52	1.5
9A9	5-10	IgG2b, κ	Ms	12,006.28	244.36	2.04	1.76
*3B2E11	6-1	IgG2a, λ	Rat	10.73	1.85	17.32	6.58
1D3G4	6-2	IgG2a, λ	Rat	NA	NA	NA	NA
EPR3194	Abcam	Unknown	Rb	16.48	1.045	6.33	10.2
	91651						

The LOD values are expressed as mean values from two independent immunoassays. Precision and uncertainty are reflected in the coefficient of variance between assays. The most sensitive antibodies are marked with stars

**Fig. 6:**
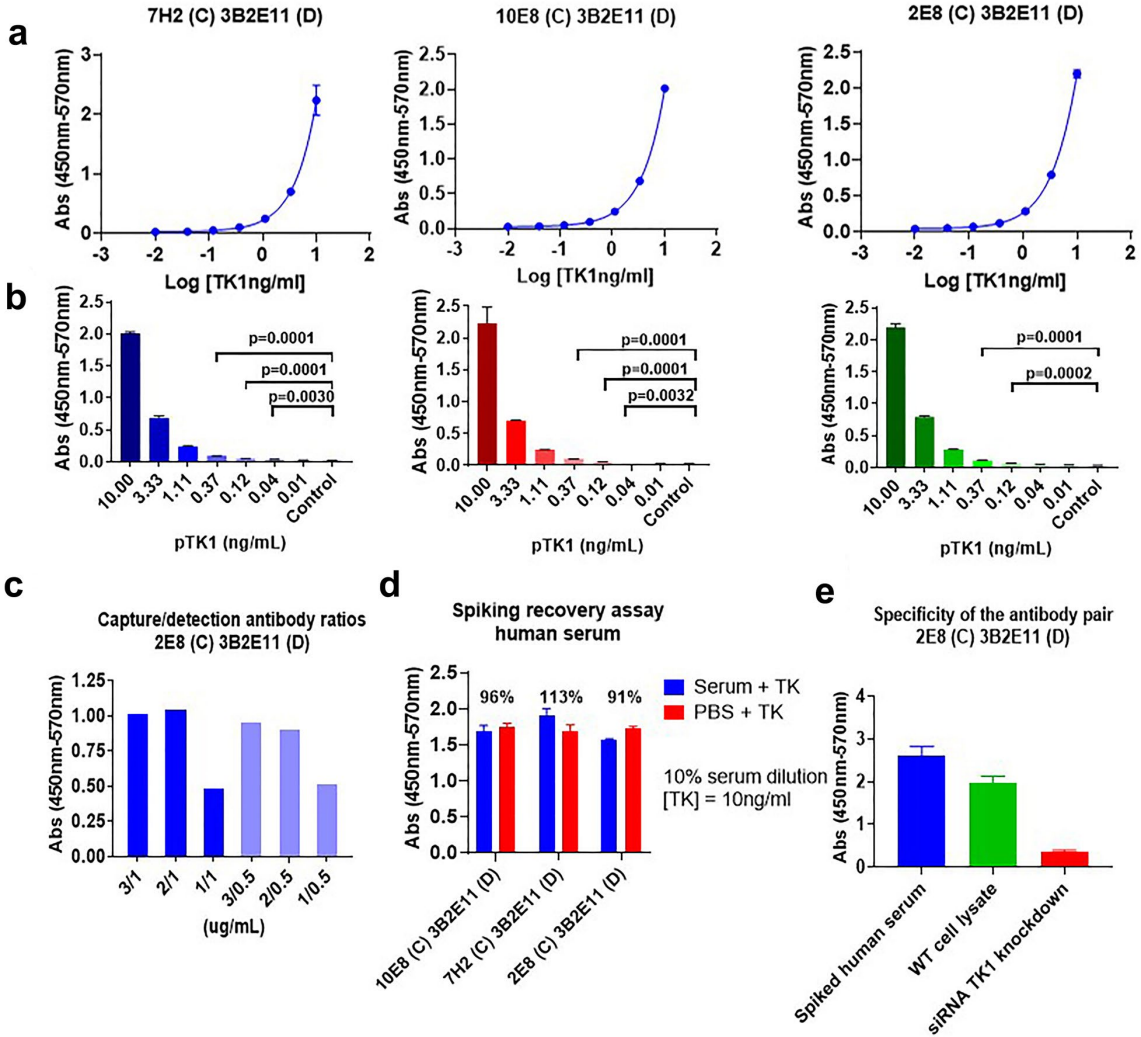
Evaluation of three antibody pairs for TK1 detection in sandwich ELISA. **a** Dose response curves and 4-point non-linear regression analysis of three antibody pairs. **b** The signal obtained from TK1 detection in serial dilutions with three antibody pairs is proportional to the concentration of antigen. **c** Optimization of capture and detection antibodies concentration ratios. **d** Spiking recovery assay. Assessment of the interference of human serum with the antibodies’ capacity to detect a known concentration of TK1. Recovery was around 100% with 10% serum dilution. **e** Specificity of the immunoassay to TK1. Using cell lysate from a high expressing TK1 cancer cell line and lysate from cells treated with siRNA TK1 the specificity of the immunoassay was evaluated. Signal from the siRNA TK1 knockdown cell lysate was significantly lower compared to the untreated cell lysate

In order to test the potential of the custom antibodies as diagnostic tools we tested different antibody pairs in sandwich ELISA. The detection antibodies were biotinylated to facilitate the union of streptavidin-HRP protein. The antibody pairs that showed consistent results were 7H2 as capture (C) and 3B2E11 as detection (D), 10E8 (C) and 3B2E11 (D) and 2E8 (C) and 3B2E11 (D) (Fig. 6a). The sensitivity of the antibody pairs ranged from 0.12 to 0.04 ng/ml, being the signal generated at these concentrations statistically significant, at least 3 standard deviations far from the blanks (Fig. 6b).

To evaluate the effect that human serum could have on the antibodies’ detection capacity, we performed a spiking recovery assay and see the ability of these three antibody pairs to detect TK1 in human serum. A 10% serum dilution was spiked with a known amount of TK1 (10 ng/ml). We then determined how much of this added TK1 could be detected expressed as a recovery percentage. For the pairs 10E8 (C) and 3B2E11 (D), 7H2 (C) and 3B2E11 (D) and 2E8 (C) and 3B2E11 (D) the recovery percentages were 96%, 113% and 91% respectively (Fig. 6d). To further test the specificity of the antibody pairs we choose the antibody pair 2E8 (C) and 3B2E11 (D) and compared the signal generated by this pair from a cell lysate positive for TK1 and a cell lysate from cells treated with siRNA TK1. Cell lysates were both diluted to have the same protein concentration. As an additional control we included spiked serum with a known concentration of TK1. We observed that there was a significant difference in the signal (about sixfold) obtained between normal cell lysate and cell lysate from cells treated with TK1 (Fig. 6e).

### Novel anti-TK1 antibodies detect TK1 surface expression in lung, breast, colon and prostate cancer cells but not on normal MNCs

**Fig. 7:**
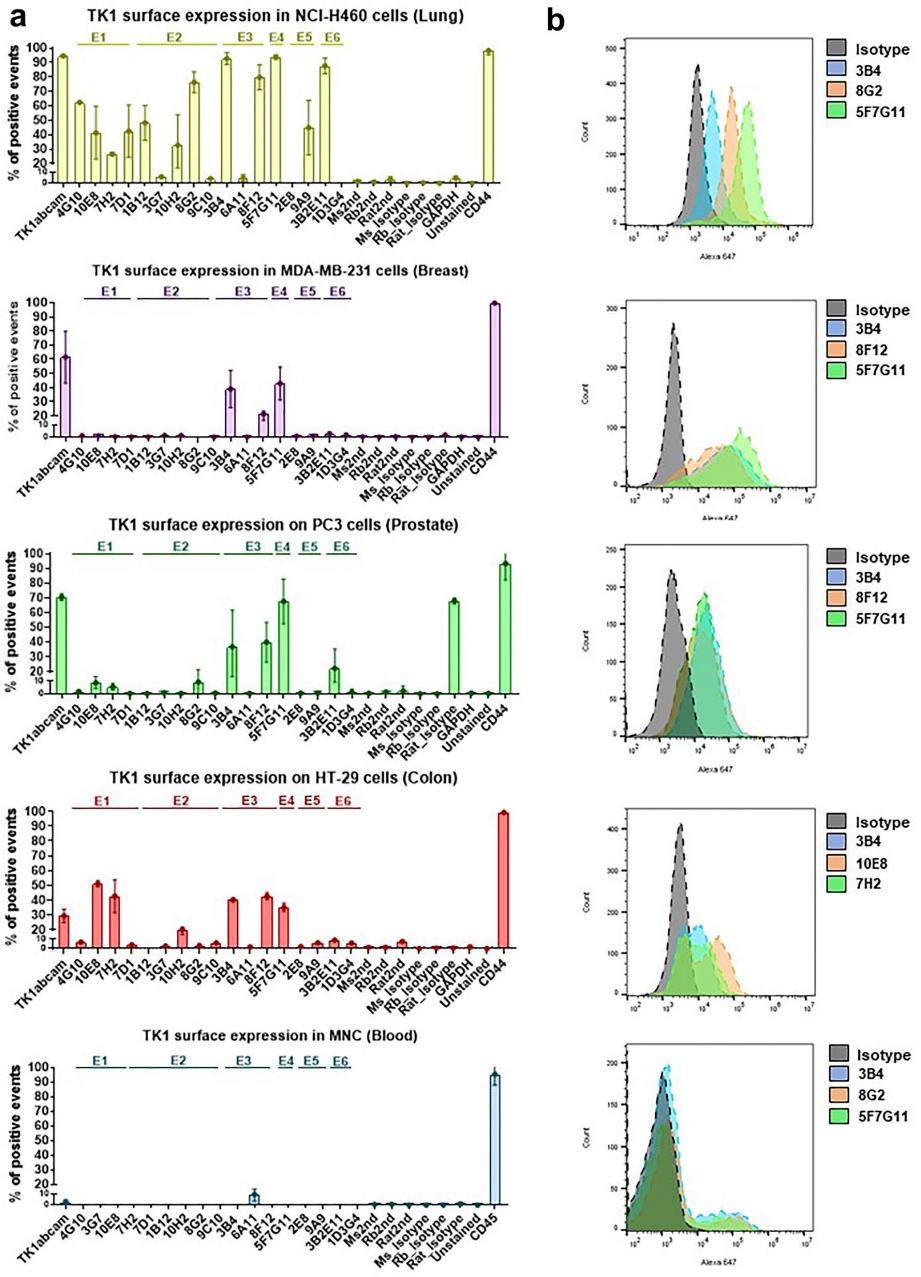
Detection of TK1 surface expression through flow cytometry. Four different cancer cell lines expressing membrane associated TK1 were stained with the custom TK1 antibodies. The binding capacities of the custom antibodies were compared to a commercial TK1 antibody. **a** The average percentage value of positive cells to TK1 from three independent flow cytometry runs across 4 cancer cell types having lung cancer cells the maximum percentage of TK1 positive cells (95.6%) followed by prostate (72.2%), colon (62.4%) and breast (49.1%). **b** Clones 3B4, 8G2, 10E8 and 7H2 showed consistent binding for detection of TK1 surface expression in lung, prostate, breast and colon cancer cell lines. In the case of H460 cells which had the highest expression levels of TK1 on the cell membrane antibodies from epitope 1 and 6 showed binding consistently. Although 5F7G11 did not showed high specificity to TK1 when tested with a TK1 knockdown the antibody showed consistent binding to the surface of all cancer cell lines were analyzed. In addition, antibody 8F12 showed consistent binding too but, did not produce any signal in indirect ELISA

During flow cytometry experiments we found that several of the anti TK1 antibodies were able to bind to membrane associated TK1 across four different cancer cell lines (Additional files 2, 3, 4, 5, 6). In the case of NCI-H460 cells, which had the highest TK1 levels, at least 1 antibody from each of the six TK1 epitopes show binding to membrane associated TK1. Although in this cancer line TK1 was targetable with all the six epitopes only epitope three seemed to be targetable on the cell membrane of all the cancer cell lines. Moreover, the expression levels of membrane associated TK1 (mTK1) changed depending on the cancer cell type. The maximum number of cells positive for membrane associated TK1 was 95.6% for NCI-H460, 72.2% for PC3, 62.4% for HT-29 and 49.1% for MDA-MB-231 cells. Two of the custom antibodies, 3B4 and 5F7G11 showed consistent binding to MDA-MB-231 (breast) and PC3 (prostate) cancer cell lines, although antibody 5F7G11 showed low affinity for TK1 in indirect ELISA (Fig. 5). This antibody also showed low specificity in antibody validation with an siRNA TK1 knockdown. Thus, we excluded this antibody as a candidate for the immunotargeting of TK1. In the case of the HT-29 (colon) cells the antibodies with the highest binding for TK1 on the cell membrane were 10E8, 7H2 and 3B4. In general, these four antibodies, 10E8, 3B4, 8G2 and 7H2 showed similar or even higher binding levels compared to antibody ab91651 and were selected as candidates to be used in ADCC experiments (Fig. 7a). None of the custom antibodies mentioned above showed significant binding to normal MNC in the case of antibody 3B2E11 that showed binding to H460 and PC3 cells we decided not to use it as candidate for ADCC experiments due to its rat isotype. In multiple times, we observed that our Rat isotype control bound to PC3 cells in similar levels as the antibody 5F7G11 which was generated in rat. Although this happened only in PC3 cells, this antibody was excluded from our subsequent antibody-dependent cell-mediated cytotoxicity (ADCC) experiments.

### TK1 can be detected in plasma membrane protein extracts from cancer cells, but not in extracts from normal cells

**Fig. 8:**
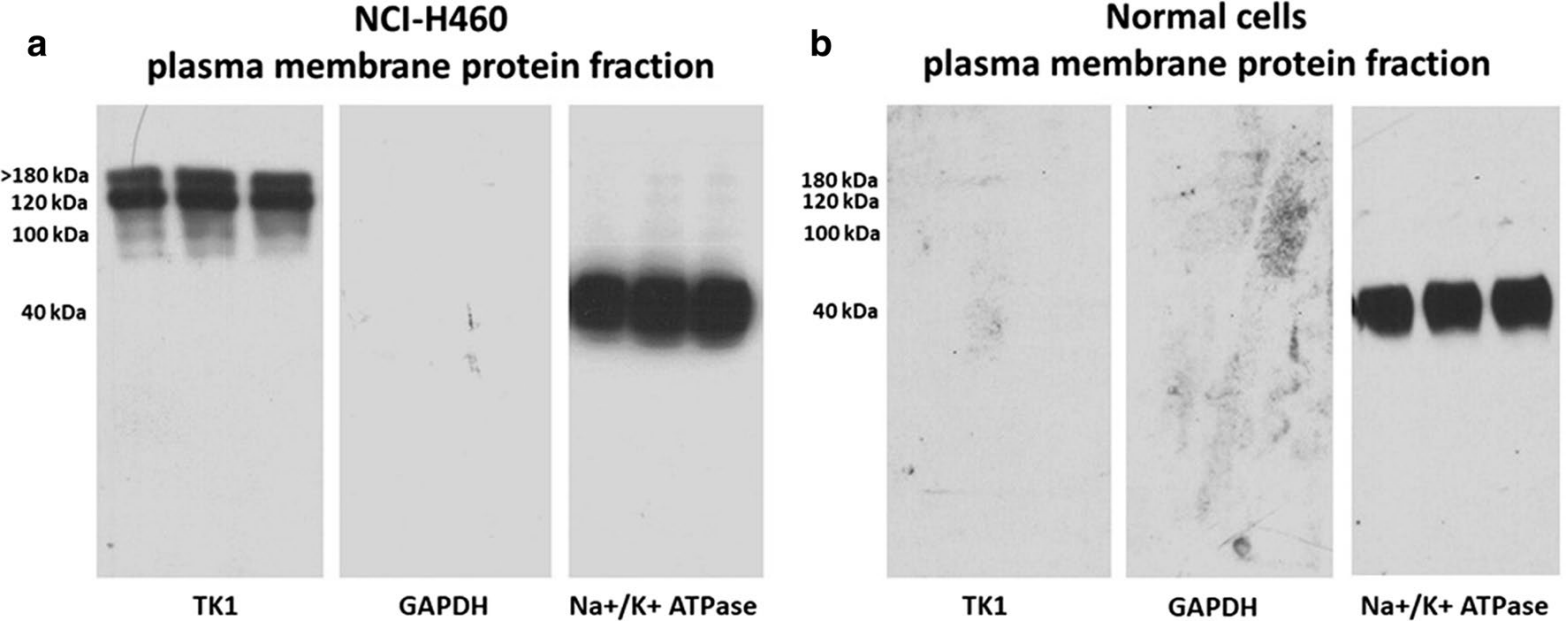
Representative western blot image for detection of membrane associated TK1 levels in plasma membrane protein extracts from the non-small cell lung carcinoma cell line NCI-H460 and healthy mono nuclear cells. **a** Plasma membrane protein extract from NCI-H460 had high levels of membrane associate TK1. **b** Membrane associated TK1 was not detected in plasma membrane proteins extracts from healthy mono nuclear cells

We found that TK1 was present in the plasma membrane protein fraction of H460 while we could not detect TK1 in the plasma membrane fraction of normal MNC cells. We did not see signal coming from the GAPDH negative control. Thus, indicating low contamination levels of cytosolic proteins in the plasma membrane protein extract. The plasma membrane protein fractions were positive for Na/K ATPase which is present on the cell membrane (Fig. 8).

### Anti-TK1 monoclonal antibodies are able to elicit ADCC responses

**Fig. 9:**
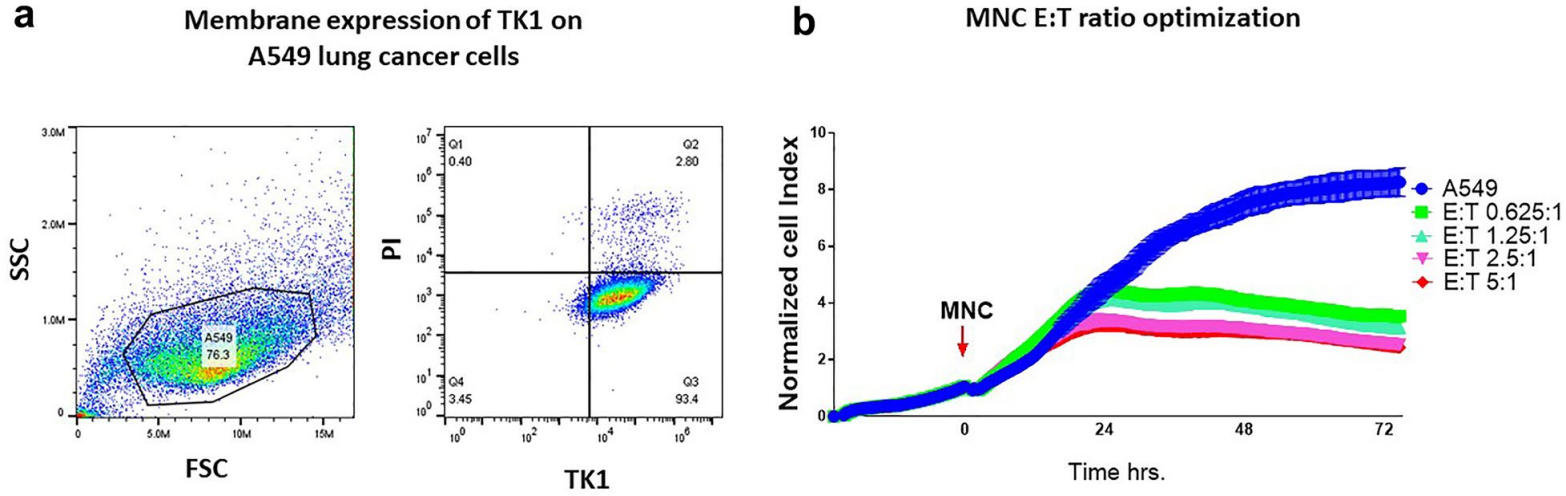
**a** Surface expression of TK1 on A549 cells detected by flow cytometry with TK1 antibody ab91651. **b** Optimization of effector and target cell ratios for ADCC assays using the ExCELLigence platform. Four different E:T ratios were tested. E:T ratios close to 1:1 showed reduced non-specific cell killing by MNCs. Eight thousand target cells (A549) were used in all wells

Since membrane expression of TK1 was detected with several of the anti-TK1 antibodies, we decided to test their potential to target TK1 and elicit an in vitro ADCC response against cancer cells. We selected the antibodies 10E8, 8G2 and 3B4 for their specificity and capacity to detect membrane expression of TK1. For these experiments two real-time cell analysis systems were used. For our initial ADCC experiments involving A549 cells we used the ExCELLigence platform. For our subsequent ADCC experiments we used the ImageXpress^®^Pico system, which allowed us to incorporate cell imaging along with our cell measurements. The A549 (lung), the MDA-MB-231 (breast) and the HT-29 (colon) cell lines used in these experiments were previously screened to confirm the expression of membrane associated TK1 (Fig. 9a).

**Fig. 10:**
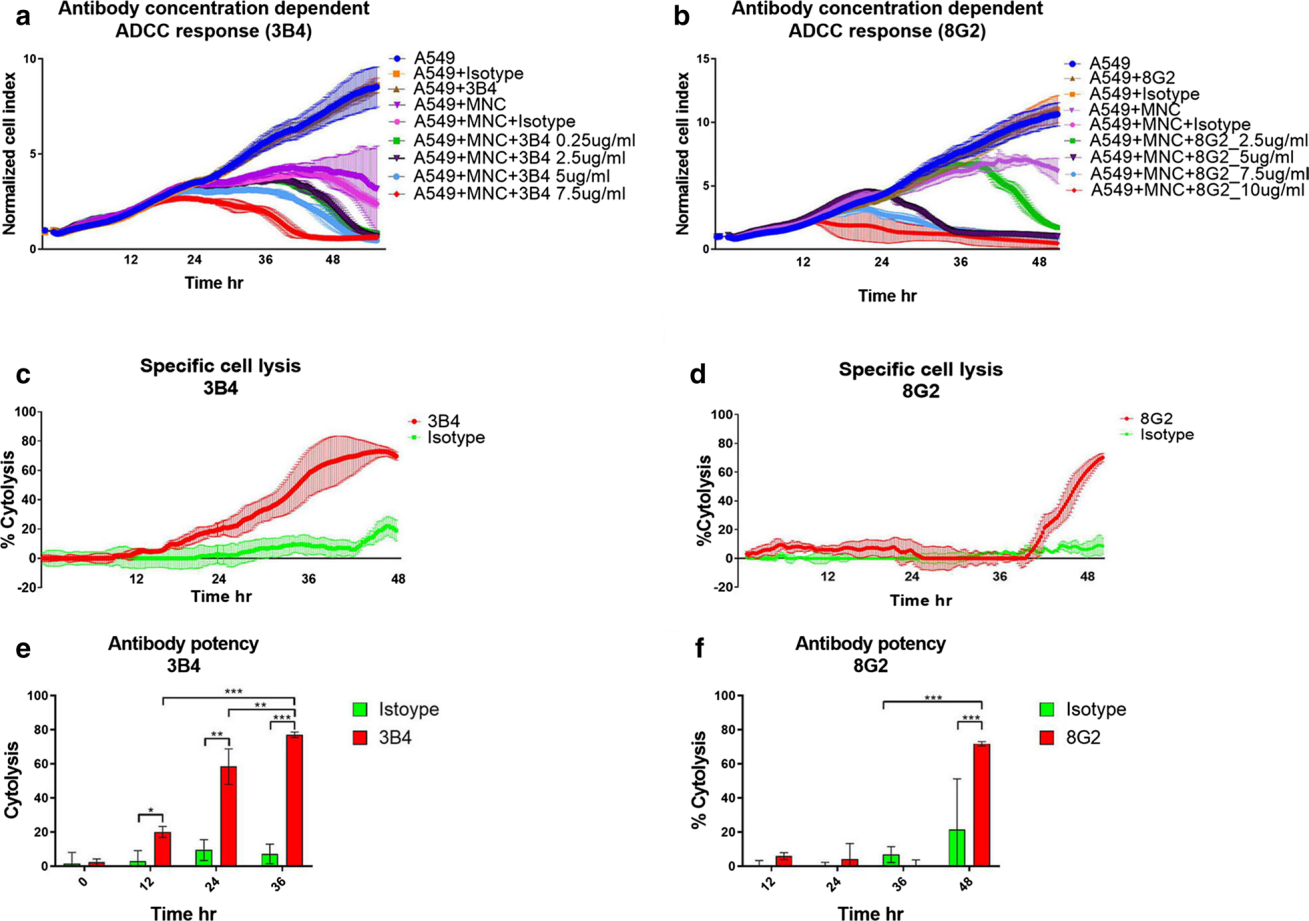
ADCC assay using TK1-specific monoclonal antibodies 3B4 and 8G2. MNC and A549 cells were co-cultured in the presence or absence of the TK1 monoclonal antibodies 3B4 and 8G2. **a** On the left a 3B4 antibody concentration dose–response curve can be observed. The ADCC response started after 24 h of treatment. **c**, **e** A significant cytolysis percentage is observed at 36 h with a 7.5 μg/ml 3B4 antibody (*p *= 0.0001). Graph **b** on the right show a dose–response curve for antibody 8G2. **d**, **f**. A significant increase in the cytolysis percentage compared to the isotype controls (*p *= 0.0003) was observed at 48 h with a 2.5 μg/ml antibody concentration

In order to find the right effector cell/target cell ratio, we first optimized the amount of effector (E) and target (T) cells without an antibody present. In the case of the ExCELLigence system we found that E:T ratios of 1.25:1 and 0.625:1 produced less background of non-specific cell killing (Fig. 9b). After finding the optimum E:T ratios, we tested different concentrations of the custom TK1 antibodies 3B4 and 8G2. The purpose of this was to find the lowest concentration of antibody at which a significant ADCC response was produced, in comparison to control groups. In the case of the antibody 3B4 (isotype IgG2b), we found that concentrations of 5.0 and 7.5 μg/ml caused significant increases in cell killing of A549 by MNC, compared to IgG2b isotype control (*p *= 0.0001) for both concentrations. In the case of the 8G2 (isotype IgG2b) antibody, a concentration of 2.5 μg/ml was enough to elicit a response significantly different from the IgG2b isotype controls (*p *= 0.0003). However, higher concentrations of 8G2 yielded significantly stronger ADCC responses (Fig. 10b). After adding the TK1 antibodies, the maximum amount of specific cell killing induced by antibodies 3B4 and 8G2 was observed at 36 h and 48 h (Fig. 10e, f). The normalized cell indexes of the target cells and effector cells were not affected by the antibody itself. Once we found the minimum concentration of antibody that was necessary to elicit a significant ADCC response we proceeded to measure the percentage of specific cytolysis. In the case of antibody 3B4, the maximum percent of cytolysis was observed after 36 h of treatment while for 8G2 antibody was observed at 48 h. An increase in specific cytolysis around 70% was observed with the antibody 8G2 (*p *= 0.001) while this value was 60% with the antibody 3B4 (*p *= 0.0001) (Fig. 10c, d).

**Fig. 11:**
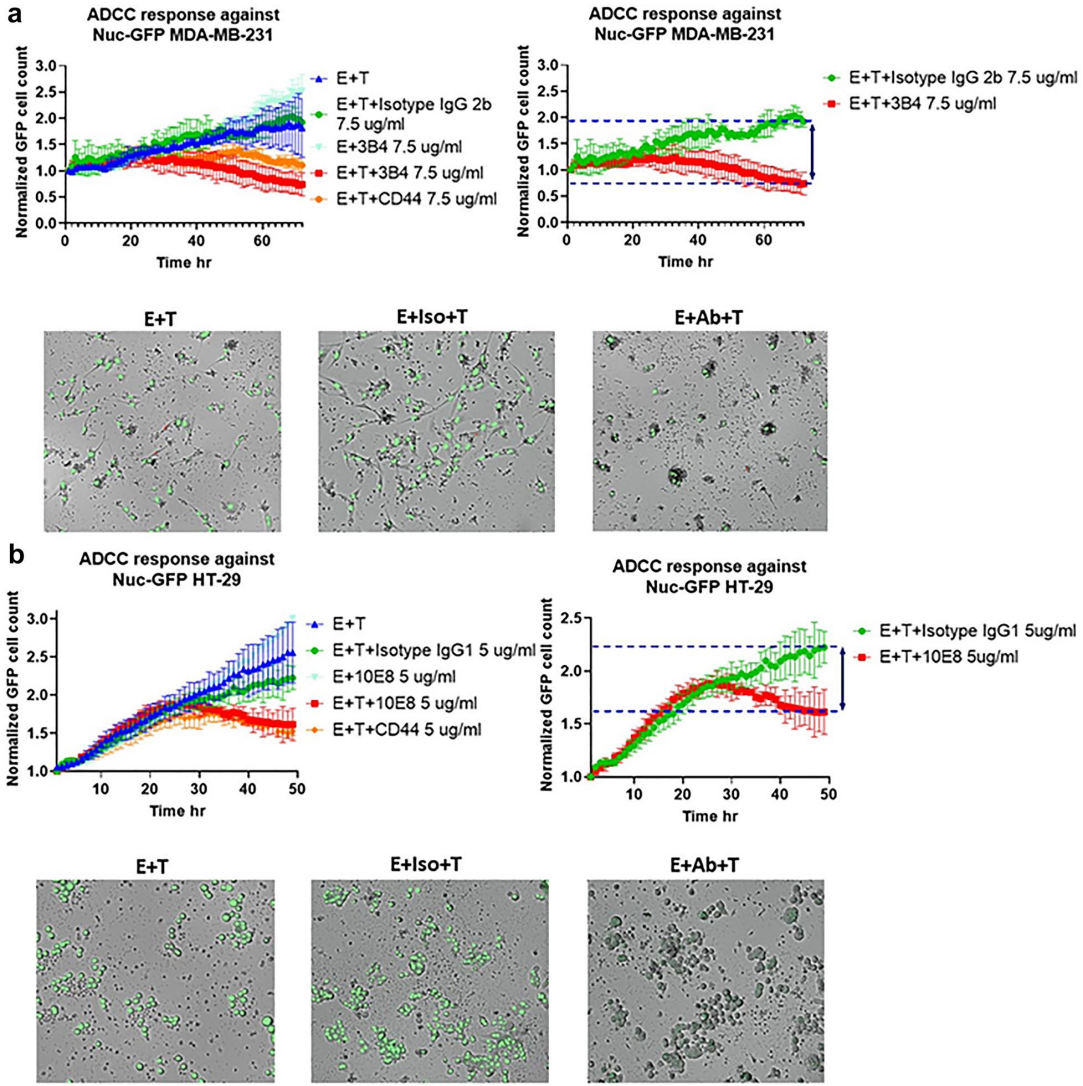
ADCC experiments on the MDA-MB231 breast cancer and the HT-29 colon cancer cell lines using the ImageXpress^®^ Pico real time cell imaging system. **a** The ADCC response against MDA-MB231 cells by MNCs using antibody 3B4. A significant decrease in the number target cells was detected at 72 h compared to the Isotype control *p *= 0.0172. **b** The ADCC response against HT-29 cell by MNCs using antibody 10E8. A decrease in the number target cells about 50% was detected at 48 h *p *= 0.0216

For our subsequent ADCC experiments we evaluated the ADCC response generated with the antibodies 3B4 and 10E8 against breast and colon tumor cells using the ImageXpress^®^ Pico system. The cell lines MDA-MB-231 and HT-29 were previously engineered to express nuc-GFP to allow us monitor cell death and cell proliferation. We found that a 5:1 E:T ration worked better for our GFP-based assays. A concentration of 5 μg/ml was enough to elicit an ADCC response with antibody 10E8 while 7.5 μg/ml was utilized for antibody 3B4. A cytolysis around 70% of the MDA-MB-231 cells was observed when antibody 3B4 was added (Fig. 11a). The ADCC response was significant when compared to the isotype control (*p *= 0.0172). In the case of HT-29 cells a cytolysis about 50% was observed using antibody 10E8 (*p *= 0.0216) which was significantly different from the isotype control (Fig. 11b). Cell imaging revealed an increased migration and clustering of MNCs around target cells when TK1 antibodies where added. Cell killing was visually confirmed by the presence of apoptotic cells and a significant reduction in the average fluorescence intensity of the target cells.

## Discussion

Scientific evidence suggests TK1 is a suitable tumor biomarker for the early detection of malignancy, tumor progression, prediction of recurrence and patient outcome [45–47]. However, new evidence indicates an emerging role of TK1 as a possible tumor target. Previous findings confirming the presence of cell membrane associated TK1 forms in tumor cells from patients, and recent studies reporting the targeting of TK1 expression inside cells have strengthened the clinical relevance of TK1 as a tumor target [23, 24]. In this study we have explored the targeting of TK1 with monoclonal antibodies. To our knowledge, this is the first time that the targeting of TK1 with monoclonal antibodies in lung, breast and colon cancer cells has been reported. In addition, we have described the generation and characterization of a new panel of high-affinity TK1 monoclonal antibodies, targeting six different epitopes, for the detection and immunotargeting of cancer.

Our results found that TK1 could be potentially targeted with antibodies at 6 different regions. In particular, antibodies against TK1 epitope two, five and six showed the highest affinities (bellow 50 pg/ml) (Fig. 5). Since these are regions that appear to be in the exterior of the tetrameric form of TK1, antibodies may have easier access to these particular epitopes. In the past TK1 has primarily been targeted towards the C-terminus and although there are some commercial antibodies that target regions at the N-terminus, there is no available data indicating that these antibodies can reliably detect TK1 in serum or membrane associated TK1 forms. To our knowledge, there is only one clinically tested TK1 immunoassay currently available and it is limited to target a region called XPA-210 towards the C-terminus [48]. Thus, the combination of antibodies targeting multiple regions of the TK1 molecule could help us increase our ability to target TK1 in cancer cells.

The antibodies potential to detect and target cancer cells was initially tested with flow cytometry (Fig. 7). We found that the antibodies developed were able to detect membrane TK1 expression in lung, breast, prostate and colon cancer cells. This may indicate the possibility of targeting a broad spectrum of tumors. In addition, we found specific epitopes that seem to be exposed in the membrane associated form of TK1. According to our flow cytometry data, the regions of the membrane associated TK1 molecule that seem to be exposed were mainly epitope two, three and four (Table 2). Epitope two is an exposed ribbon in the tetrameric form of TK1 located towards the N-terminus while epitope three is a ribbon towards the center of the molecule. Epitope four contains a lasso and the active site of the molecule, suggesting that membrane associated TK1 may keep kinase activity [23]. However, depending on which cancer cell line was analyzed we observed that other epitopes were exposed as well. This was the particular case for H460 and HT-29 cells where anti TK1 antibodies for epitopes one and six showed binding to the cell membrane, while these epitopes were not detectable in MB-MDA-231 cells and PC3 cells. It is important to mention that NCI-H460 cells where the cells with the highest levels of TK1 on the cell membrane. Thus, it is possible that the higher expression levels of surface TK1 make these epitopes more accessible for antibodies. The absence of significant binding of the anti TK1 antibodies to normal cells confirms our previous reports that TK1 membrane expression may be restricted to malignancy. This was further confirmed by Western blot analysis of membrane associated TK1 protein levels in NCI-H460 cells plasma membrane protein extracts and plasma membrane protein extracts from normal MNCs (Fig. 8). Thus, making TK1 an immunotarget with low off-target effects that could be used for antibody-based or cell adoptive therapies such as chimeric antigen receptor (CAR) T cell therapy [49]. Furthermore, if membrane expression of TK1 is restricted to malignant cells, then the detection of membrane associated TK1 forms may be a hallmark linked to the development of particular cancers.

Although the role of TK1 as a tumor biomarker has extensively been studied, little has been done to explore the potential of TK1 as tumor target. Previous studies have suggested that TK1 in malignant cells may be different than TK1 in normal cells. In fact, the targeting of malignant forms of TK1 was suggested almost three decades ago by other research groups [21, 22]. Due to their high specificity monoclonal antibodies are ideal candidates for the specific targeting of TK1 in cancer cells. In addition, we did not find any available studies about targeting TK1 with monoclonal antibodies particularly targeting its membrane associated forms in malignant cells. Thus, our ADCC experiments were designed to explore this possibility.

During this study three of our custom TK1 antibodies, antibodies 10E8 (targets epitope one), 8G2 (targets epitope 2) and 3B4 (targets epitope three) were selected for their specificity and capacity to detect membrane associated TK1 on malignant cells. In this case the anti TK1 antibodies’ ability to elicit an ADCC response was measured. It is important to mention that even though the commercial anti-TK1 antibody ab91651 could detect surface expression of TK1 its particular rabbit isotype made it unsuitable for our ADCC experiments. We found a significant increase in the cytolysis of lung, breast and colon cancer cells by MNC when the anti-TK1 antibodies were added in comparison to controls (Figs. 10 and 11). Thus, confirming previous reports of the membrane expression of TK1 and opening the door for additional studies exploring the targeting of TK1 in cancer cells.

The mechanisms through which TK1 is expressed on the surface of cancer cells remains unknown. However, there are some indications of its associations with the cell membrane. In the past other pyrimidine salvage pathway enzymes were found to be associated to the membrane of cancer cells [50]. Other studies using yeast-two hybrid experiments have found TK1 interacting with other membrane proteins such as SEZL6 that are upregulated in lung cancer cells [51, 52]. Therefore, the characterization of novel TK1 protein–protein interactions on the membrane of cancer cells can possibly lead to the development of more specific targeted therapies. The dual targeting of tumor antigens is a feasible strategy that has been tested before in cancer immunotherapy [53].

The experiments presented here provide early evidence that the targeting of membrane associated TK1 with monoclonal antibodies may be a feasible approach for the treatment of cancer. In addition, we have developed a novel panel of TK1 antibodies that may be useful for TK1-based diagnostics. Future directions of this research will focus on the targeting of several other cancer cell lines, In vivo ADCC experiments and the development of both antibody-based and cell adoptive therapies targeting TK1.

## Conclusions

The antibodies presented here had the ability to detect and quantify TK1 in the picomolar range and potentially could allow us to measure TK1 levels in cancer patients. In addition, the antibodies were able to detect multiple forms of TK1 including membrane associated TK1 in lung, breast, colon and prostate cancer cells. Thus, TK1 may be a tumor target that can be used for therapy in multiple solid malignancies. The increased cytolysis of A549, MDA-MB-231 and HT-29 cells by MNCs in the presence of anti TK1 monoclonal antibodies during the in vitro ADCC experiments suggests that, TK1 monoclonal antibodies have potential not only as diagnostic tools but also as immunotherapeutic agents for the treatment of cancer. Therefore, the immunotargeting of TK1 may be a feasible approach for the elimination of cancer cells. The exploration of TK1 as a tumor target may lead to the development of other TK1-based immunotherapeutics.

## Supplementary information

**Additional file 1.** Production and validation of human recombinant TK1 in a yeast-based expression system.

**Additional file 2.** Flow cytometry analysis of NCI-H460 cells with the custom TK1 antibodies.

**Additional file 3.** Flow cytometry analysis of MDA-231 cells with the custom TK1 antibodies.

**Additional file 4.** Flow cytometry analysis of PC3 cells with the custom TK1 antibodies.

**Additional file 5.** Flow cytometry analysis of HT-29 cells with the custom TK1 antibodies.

**Additional file 6.** Flow cytometry analysis of normal MNC cells with the custom TK1 antibodies.

## Data Availability

Data files generated and/or analyzed during the current study are available from the corresponding author upon reasonable request.

## Abbreviations

TK1: Thymidine kinase 1
MNC: Mono nuclear cells
ELISA: Enzyme-linked immunosorbent assay
LOD: Limit of detection
LOQ: Limit of quantification
ADCC: Antibody-dependent cell-mediated cytotoxicity
