# Supplementary material for: Novel monoclonal antibodies against thymidine kinase 1 and their potential use for the immunotargeting of lung, breast and colon cancer cells
Source: Cancer Cell Int. 2020 Apr 16;20:127. doi: 10.1186/s12935-020-01198-8 (PMC7160906; doi:10.1186/s12935-020-01198-8)

**Additional file 1.** Production and validation of human recombinant TK1 in a yeast-based expression system. The sequence of human TK1, transcript variant 1, (Accession No. NM_003258.5) was codon optimized to be expressed in a *Saccharomyces cerevisiae* yeast strain with a REG-1 mutation. A 6xHis tag was added at the C-terminus to facilitate His-tag purification. The optimized TK1 sequence was synthesized and cloned into the pESC-URA vector (Genscript, Piscataway, NJ) and was purified by affinity chromatography using NI-NTA-agarose beads columns (Qiagen, Hilden, Germany). In house produced TK1 was validated along with commercially outsourced recombinant TK1 in Western blot using the anti-TK1 antibody ab91651 (Abcam, Cambridge, UK).


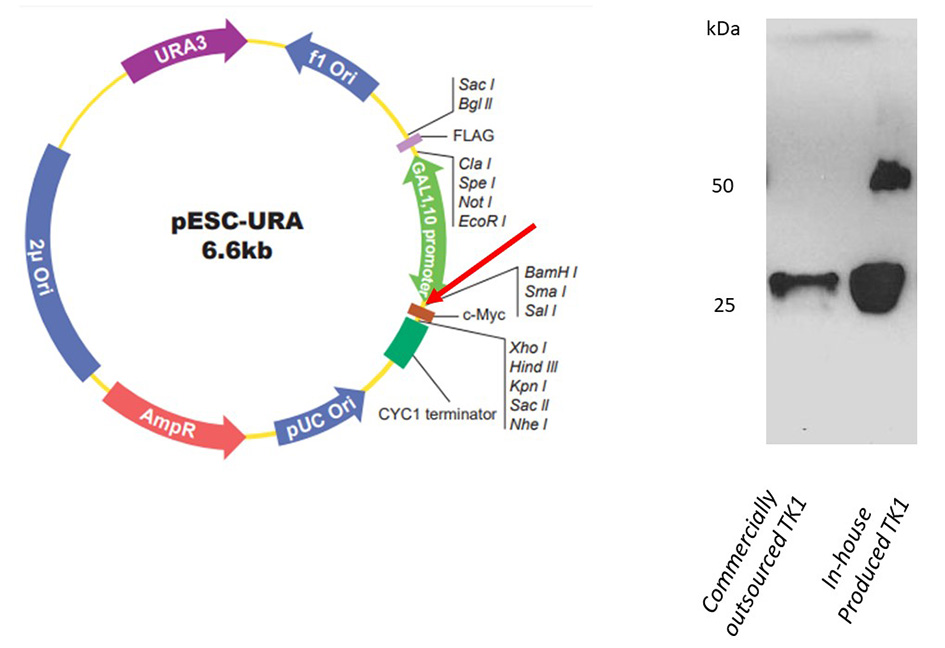

Supplement: Supplementary file 1 — Additional file 1. Production and validation of human recombinant TK1 in a yeast-based expression system. [file 12935_2020_1198_MOESM1_ESM.docx]
