# Supplementary material for: Novel monoclonal antibodies against thymidine kinase 1 and their potential use for the immunotargeting of lung, breast and colon cancer cells
Source: Cancer Cell Int. 2020 Apr 16;20:127. doi: 10.1186/s12935-020-01198-8 (PMC7160906; doi:10.1186/s12935-020-01198-8)

**Additional file 6**. Flow cytometry analysis of normal MNC cells with the custom TK1 antibodies. See Table 3 to find the name of each clone associated to its respective ID.


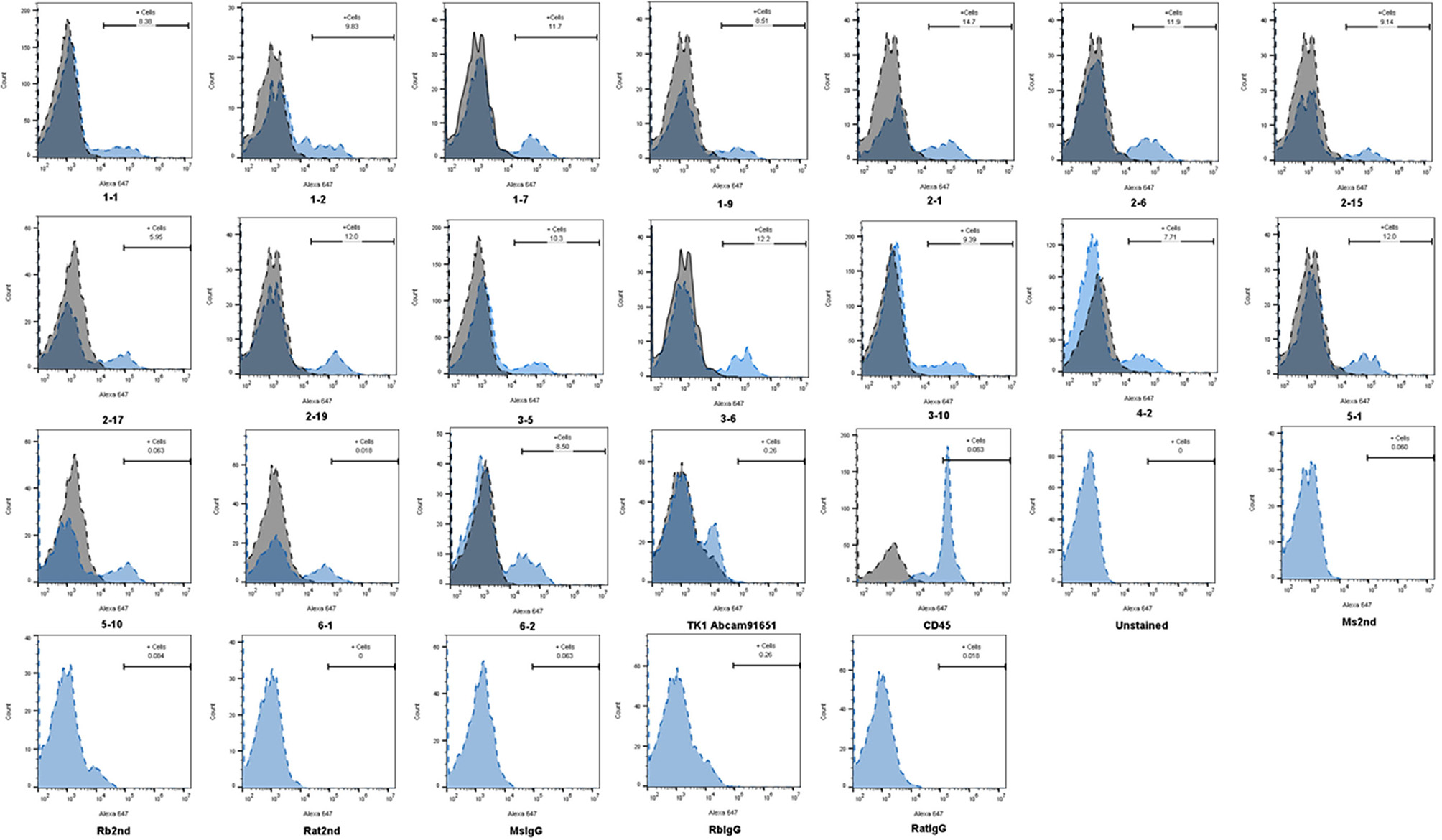

Supplement: Supplementary file 6 — Additional file 6. Flow cytometry analysis of normal MNC cells with the custom TK1 antibodies. [file 12935_2020_1198_MOESM6_ESM.docx]
